# Cardiovascular disease risk assessment among patients attending two cardiac clinics in the Ashanti Region of Ghana

**DOI:** 10.4314/gmj.v54i3.3

**Published:** 2020-09

**Authors:** Waindim Nyiambam, Augustina A Sylverken, Isaac K Owusu, Kwame O Buabeng, Fred A Boateng, Ellis Owusu-Dabo

**Affiliations:** 1 Department of Community Health, Kwame Nkrumah University of Science and Technology, Kumasi, Ghana; 2 Kumasi Centre for Collaborative Research in Tropical Medicine (KCCR), Kwame Nkrumah University of Science and Technology, Kumasi, Ghana; 3 Department of Medicine, Kwame Nkrumah University of Science and Technology, Kumasi, Ghana; 4 Department of Clinical and Social Pharmacy, Kwame Nkrumah University of Science and Technology, Kumasi, Ghana; 5 Suntreso Government Hospital, Kumasi, Ghana

**Keywords:** cardiovascular diseases, hypertension, Kumasi, total risk, Framingham risk score

## Abstract

**Background:**

Cardiovascular disease (CVD) is a major cause of morbidity and hypertension is the single most important modifiable risk. Assessment of an individual's “total” predicted risk of developing a CVD event in 5- or 10-years using risk scores has been identified as an accurate measure of CVD risk. Using the latest Framingham risk score we assessed the risk among patients attending two cardiac clinics in Kumasi.

**Methods:**

We conducted a hospital-based cross-sectional study among 441 patients attending two cardiac clinics in Kumasi, the Ashanti region of Ghana. Hospital records were reviewed and information on demography, social history and laboratory results for the lipid profile tests were extracted.

**Results:**

The prevalence of low, medium and high risk were 41.5%, 28.1% and 30.4% respectively. More men were at high risk compared to females (36.0% vs 23.9%, p=0.003). The risk score showed good discrimination for cardiovascular risk stratification with an overall area under the curve of 0.95; 0.97 and 0.94 for males and females respectively. The sensitivity and specificity of the Framingham risk score were 89.5% and 86.3%, respectively.

**Conclusion:**

Majority of our study participants were at moderate to high risk with men being the most affected. The Framingham risk score proved to be a useful tool in predicting the 10-year total cardiovascular disease risk.

**Funding:**

Not indicated

## Introduction

Current epidemiological evidence indicates that cardiovascular disease (CVD) largely contributes to the non-communicable diseases (NCDs) burden in low and middle-income countries.^1^ Globally, an estimated 17.3 million people died from CVDs in 2008 with over 80% of the deaths taking place in low and middle-income countries.

Projections indicate that mortality from NCDs will increase in all regions of the world over the next decade with the greatest increase expected to occur in Africa^1^ where there has been an epidemiological transition from predominantly infectious diseases to NCDs.

In Ghana, CVD was evidenced as the number one cause of death in 1991 and 2001 and has remained as the major cause of mortality in the country.^2^

The major risk factors for CVD particularly hypertension and their impact are known and remain similar in most regions of the world.^3^ These include the changing demographic profile with a greater survival into adulthood and relative ageing of the population. Others are unplanned urbanization and changes in lifestyle associated with economic development which includes diet, smoking, adiposity and alcohol use.^1^,^4^

Even in the face of these similar and well-known determinants, while many high-income countries are experiencing a decrease in the prevalence of risk factors, it is the contrary in many low- and middle-income countries where a rise in the prevalence of risk factors has been reported by several studies.^5^–^10^

Risk factors have been shown to cluster and interact synergistically to promote CVD and epidemiological evidence indicates that combining risk factors into scores enables an individual's ‘total’ cardiovascular risk to be predicted with reasonable accuracy.^11^–^15^ Total cardiovascular disease risk is the probability of an individual experiencing a cardiovascular event over 10 years using the most recent Framingham risk scoring algorithm.^13^

Risk scoring, therefore, makes individuals become aware of their risk status and can therefore serve as enough motivation for engaging in activities to lower overall risk. This is because CVD events are noted to develop gradually and silently. As such, most people are not aware of their risk status and may fail to optimize and individualize potential preventive strategies. Additionally, it can potentially provide a means of making decisions about intervening in a targeted way, thereby making use of resources available to reduce risk in developing the disease.^1^ Furthermore, given that CVD risk increases with age, as life expectancy increases in most low and middle income countries such as Ghana, it is expected that the number of people who will experience a CVD event will increase significantly in the near future.^16^

It is, therefore, imperative that urgent measures are taken to know the burden of CVD risk. Even though some few studies have assessed the prevalence of individual risk factors of CVD in Kumasi^10^, there is no information on the effect of the combination of risk factors on total cardiovascular risk in the region. In order to devise strategies to reduce risks, optimize health outcomes for CVDs, we assessed the total cardiovascular disease risk amongst persons attending two Cardiac Clinics in the Ashanti Region of Ghana.

## Methods

### Study area

We conducted our study at the cardiac clinics of the Komfo Anokye Teaching Hospital (KATH), and Precise Specialist Clinic Amakom, both in Kumasi, Ghana. KATH is a 1000-bed capacity tertiary medical institution that receives referrals from eight of the ten regions of the country owing to its strategic location at the confluence of the country's transportation network.

The Precise Specialist Clinic at Amakom in Kumasi is a recognized specialized private cardiac clinic that attends to and follows up patients with cardiovascular diseaserelated conditions.

### Study design, inclusion/exclusion criteria and definitions

Our study was a hospital-based cross-sectional study at the two sites. Hospital-based records of patients 20 years and above who attended our study clinics from January 2012 to July 2014 were reviewed. Apart from demographic data, information on systolic blood pressure, diastolic blood pressure, diabetes mellitus, body mass index (BMI), alcohol consumption, smoking and hypertension were taken.

We defined hypertension as the presence of persistent elevated systolic blood pressure ≥140mmHg and/or diastolic blood pressure ≥90mmHg and/or use of antihypertensive drugs. A participant with past medical history of hypertension was also classified as being hypertensive^17^. Diabetes mellitus was defined as a random blood glucose level ≥11.1mmol/l and/or fasting blood glucose level ≥7.0 mmol/l and/or use of insulin or an oral hypoglycaemic agent.^18^ We computed the BMI ((kg/m^2^) of our study participants by dividing their weights in kilograms by the square of their heights in meters. Obesity was defined as BMI ≥30kg/m^2^. We also defined overweight as those with BMI between 25.00 and 29.99kg/m^2^. Normal weight was a BMI ranging between 18.5 and 24.99kg/m^2^.

Clinical diagnosis and laboratory results of fasting blood sugar, total cholesterol, high density lipoprotein cholesterol (HDL-C), low density lipoprotein cholesterol (LDL-C), triglycerides as at the last visit were collected and recorded on data capture sheets. All information obtained was used to calculate the 10-year total cardiovascular risk using the most recent Framingham risk scoring algorithm.^13^

We excluded patients whose records showed established or a history of any cardiovascular disease. Established CVDs constituted any diagnosis of Coronary Heart Disease (heart attack), Cerebrovascular Disease (stroke), Peripheral Arterial Disease, Pulmonary Embolism, deep vein thrombosis, Congenital Heart Disease and Rheumatic Heart Disease by a Cardiologist.

Additionally, all those with missing values for any covariate were omitted from our study. Finally, we also excluded those whose records did not show regular attendance from January 2012. A regular attendance was based on documentation of blood pressure readings, diagnosis, prescriptions, laboratory tests ordered or appointments by the cardiologists.

Absence of any of these from 2012 till the time the study was conducted was considered irregular attendance’.

### Data analysis

STATA version 11.1 (STATA CORPORATION, College Station, Texas, USA) was used to analyze the data. Percentages were used to describe categorical variables while mean ± standard deviation was used for continuous variables. For comparison of risk factors between men and women, the chi-square test was used for categorical variables and independent t-test for continuous variables. The test for trend was used to determine the trend of the relationship between covariates and CVD risk. Univariate and multivariate ordered logistic regression were used to determine the effect covariates on CVD risk. The discrimination of the model (ability of the model to classify those with moderate to high risk) was assessed using the receiver operating characteristic (ROC) curve. A value of >0.75 was considered good discrimination. For all analyses p-values less than 0.05 were considered statistically significant.

### Ethics, consent and approval

We obtained ethical approval from the Committee on Human Research Publication and Ethics (CHRPE) of the School of Medical Sciences, Kwame Nkrumah University of Science and Technology, Kumasi and Komfo Anokye Teaching Hospital. We also sought permission from the individual heads of our study hospitals and as well as obtained consent from individuals who agreed to have their records used in the study.

## Results

### Characteristics of the study population

Overall, there were 441 participants with half of them from each site. There were more males (53.5%) than females. The average age was 54.35 (sd±12.9) years with females having a significantly higher age (p=0.004). The mean body mass index (BMI) was 30.26 (sd±5.94) and females had a significantly higher BMI (p<0.001).

Significantly more males (44.5%) were overweight compared to females (28.3%) (p<0.001). However, more females turned out to be obese (61.9% females compared to 31.8% males) (p<0.001). A little over one-third of the study participants had been hypertensive for less than ten years. Apart from total cholesterol and LDL-C which showed significant differences between males and females (p=0.02), the mean values of most of the main clinical CVD risk factors were slightly raised or borderline compared to clinical thresholds. Characteristics of the study population are shown in Table 1.

**Table 1:** Characteristics of the study population

Variable	Overall (n=441)	Male (n=236)	Female (n=205)	p-value
**Age (years), mean** **(sd)**	54.35 (12.90)	52.69 (12.47)	56.26 (13.15)	**0.004**
**BMI(kg/m^2^), mean** **(sd)**	30.26 (5.94)	28.44 (5.07)	32.36 (6.17)	**<0.001**
**BMI category, n** **(%)**				
**Normal weight**	76 (17.20)	56 (23.70)	20 (9.80)	
**Overweight**	163 (37.00)	105 (44.50)	58 (28.30)	**<0.001**
**Obese**	202 (45.80)	75 (31.80)	127 (61.90)	
**Hypertension duration** **in years, n** **(%)**				
**<10**	334 (75.70)	179 (53.60)	155 (46.40)	
**10 – 19**	73 (16.50)	41 (56.20)	32 (43.80)	
**20 – 29**	26 (6.00)	12 (46.20)	14 (53.80)	0.846
**≥ 30**	08 (1.80)	4 (50.00)	4 (50.00)	
**Treatment of hypertension,** **n (%)**	269 (61.00)	149 (55.40)	120 (44.60)	0.32
**Systolic BP** **(mmHg) mean (sd)**	137.66 (21.44)	138.02 (21.73)	137.23 (21.15)	0.69
**Diastolic BP** **(mmHg) mean (sd)**	76.38 (14.09)	77.25 (15.09)	75.38 (12.75)	0.16
**TC (mmol/l) mean** **(sd)**	5.19 (1.25)	5.05 (1.20)	5.34 (1.29)	**0.02**
**Triglycerides** **(mmol/l) mean (sd)**	1.35 (0.62)	1.32 (0.63)	1.39 (0.61)	0.26
**HDL-C (mmol/l)** **mean (sd)**	1.23 (0.37)	1.22 (0.36)	1.23 (0.37)	0.65
**LDL-C (mmol/l)** **mean (sd)**	3.32 (1.04)	3.21 (0.99)	3.44 (1.08)	**0.02**
**FBS (mmol/l) mean** **(sd)**	5.81 (1.88)	5.75 (1.78)	5.87 (2.00)	0.5

BP-blood pressure; TC–Total cholesterol; HDL-C-high-density lipoprotein cholesterol; LDL-C-low density lipoprotein cholesterol; FBS-fasting blood sugar Bold font denotes significance

### Prevalence and distribution of Cardiovascular Risk

Our results revealed that of the total risk factors, hypertension was the commonest (52.6%), followed by obesity (33.3%) and diabetes mellitus (12.4%). Only men were found to have a history of cigarette smoking.

More men had moderate to high CVD risk (p=0.003). As age increased so did CVD risk and the trend was significant (p<0.001). From age 45 through >70 years increasingly more people were at higher risk. The majority of people who were hypertensive, diabetic and had a history of cigarette smoking were at moderate to high CVD risk. There was no significant increase in CVD risk with increasing BMI (p=0.492) and alcohol consumption (p=0.82). There was a significant increase in CVD risk as the levels of lipids increased, but for the triglycerides, the trend was only marginal (p=0.057). As the duration of hypertension increased so did CVD risk (p<0.0001).

The majority of participants who were on antihypertensive medications had moderate to high CVD risk (Table 2).

**Table 2:** Distribution of 10-year CVD risk

Variable	Level of CVD RISK			p-value
	Low	Moderate	High	
**Sex**				
**Male**	85 (36.0)	66 (28.0)	85 (36.0)	p=0.003
**Female**	98 (47.8)	58 (28.3)	49 (23.9)	
**Age group (years)**				
**< 30**	9 (100.0)	0 (0.0)	0 (0.0)	
**30 – 34**	23 (100.0)	0 (0.0)	0 (0.0)	
**35 – 39**	25 (96.5)	1 (3.5)	0 (0.0)	
**40 – 44**	22 (73.3)	8 (26.7)	0 (0.0)	
**45 – 49**	40 (67.8)	18 (30.5)	1 (1.7)	
**50 – 54**	29 (36.3)	38 (47.5)	13 (16.2)	pt<0.0001
**55 – 59**	17 (30.4)	21 (37.5)	18 (32.1)	
**60 – 64**	5 (10.2)	14 (28.6)	30 (61.2)	
**65 – 69**	7 (18.0)	11 (28.2)	21 (53.8)	
**≥ 70**	3 (4.5)	13 (19.4)	51 (76.1)	
**Hypertension**				
**No**	92 (75.4)	19 (15.6)	11 (9.0)	p<0.0001
**Yes**	91 (28.5)	105 (32.9)	123 (38.6)	
**Diabetes**				
**No**	180 (49.2)	107 (29.2)	79 (21.6)	p<0.0001
**Yes**	3 (4.0)	17 (22.7)	55 (73.3)	
**Smoking**				
**No**	181 (42.1)	122 (28.4)	127 (29.5)	p=0.027
**Yes**	2 (18.2)	2 (18.2)	7 (63.4)	
**Treatment of hypertension**				
**No**	113 (65.7)	38 (22.1)	21 (12.2)	
**Yes**	70 (26.0)	86 (32.0)	113 (42.0)	p<0.0001
**Hypertension duration (years)**				
**< 10**	168 (50.3)	92 (27.5)	74 (22.2)	
**10–19**	12 (16.5)	19 (26.0)	42 (57.5)	
**20–29**	3 (11.5)	10 (38.5)	13 (50.0)	p<0.0001
**≥ 30**	0 (0.0)	3 (37.5)	5 (62.5)	

Pt-p-value for trend

### Association Between CVD Risk Factors and 10-year CVD Risk

Our results indicated that women were about 41% less likely to be at high CVD risk compared to moderate risk. For a one-unit increase in age (5 years) the odds of having high risk compared to moderate risk increased by about 2. The odds of having high risk compared to moderate risk was 11.02 times greater for those who had diabetes. For all lipids but for triglycerides, the odds of having high CVD risk compared to moderate risk was not significantly greater (or lower for HDL-C) for those who had borderline levels compared to those whose levels were optimal. People who smoked cigarette were roughly 4 times more likely of having high risk compared to moderate CVD risk.

Multivariate analysis indicated that women were 94% less likely to be at high risk compared to men for moderate risk, holding all other predictors constant.

For each one-unit (5 years) increase in age, the odds of having high risk compared to moderate risk increased to about 3.7 adjusting for other covariates. However, large odds ratios and confidence intervals observed. After controlling for other covariates, total cholesterol and triglyceride were no longer significant but were included in the final model because they are important predictors of CVD risk (Table 3).

**Table 3:** Association between CVD risk factors and 10-year CVD risk

Variables	Crude OR (95% CI)	p-value	Adjusted OR (95% C I)	p-value
**Sex**				
**Male**	1		1	
**Female**	0.59 (0.42–0.84)	0.003	0.06 (0.03–0.13)	<0.001
**Age group** **(years)**	2.18 (1.94–2.45)	<0.0001	3.70 (2.99–4.57)	<0.001
**Hypertension**				
**No**	1		1	
**Yes**	7.43 (4.66–11.87)	<0.0001	20.53 (9.53–44.25)	<0.001
**Diabetes**				
**No**	1		1	
**Yes**	11.02 (6.32–19.21)	<0.0001	68.94(27.38–173.6)	<0.001
**Total cholesterol**				
**Optimal**	1		1	
**Borderline** **high**	1.18 (0.79–1.76)	0.42	0.94 (0.38–2.37)	0.905
**High**	2.5 (1.54–4.05)	<0.0001	2.95 (0.87–10.03)	0.083
**Triglycerides**				
**Optimal**	1		1	
**Borderline** **high**	1.84 (1.21–2.8)	0.004	1.21 (0.61–2.36)	0.585
**High**	1.11 (0.56–2.21)	0.75	1.67 (0.55–5.00)	0.361
**Smoking**				
**No**	1		1	
**Yes**	3.96 (1.17–13.44)	0.027	17.57 (2.48–124.09)	0.004
**Alcohol use**				
**No**	1			
**Yes**	1.12 (0.45–2.78)	0.81		
**Hypertension Treatment**				
**No**	1			
**Yes**	5.37 (3.63–7.96)	<0.0001		
**Hypertension** **duration**	2.57 (1.91–3.44)	<0.0001		

### Sensitivity and specificity of the Framingham risk score

The model showed good discrimination overall and for both sexes at the cut off of 20% for moderate to high risk for this study. It correctly stratified 85.3% study participants overall into low and moderate/high risk; 86.8% men and 86.0% women.

## Discussion

Over the years, cardiovascular diseases (CVDs) have risen to be among the top causes of admission and institutional deaths in Ghana. This rise parallels a rise in risk factors. Assessment of an individual's “total” predicted risk of developing a CVD event in 5- or 10-years using risk scores has been identified as one of the ways to determine the burden of CVD risk and to guide treatment decisions.

Results of our study indicated that more than half of our study participants were at moderate to high 10-year CVD risk. This is similar to findings of a hospital-based study in Malaysia which also used the Framingham risk score^19^ and in Bulgaria^20^ where the SCORE risk algorithm was used. Our finding is however contrary to studies in Cuba^19^, three middle income countries in Asia^21^ and Jamaica^22^ where the majority of the population (>90%) was classified as low risk. This is probably because these contradicting studies besides being population-based studies rather used the WHO/ISH risk scoring charts to estimate total CVD risk. Apart from not being calibrated and validated in most countries^12^, the WHO/ISH risk scoring charts have been shown in comparative studies to classify majority of individuals as low risk despite the high prevalence of CVD risk factors in those populations^19^ and 23.

There was a significant sex difference in the distribution of the 10-year CVD risk, with males being more likely to be in the moderate to high risk categories even though women were significantly older than men. Our finding is in contrast with that of a study in Jamaica^22^ in the distribution of 10-year CVD risk. It however agrees with a study in Bulgaria where men had a significantly higher risk than women.^20^ The decreased likelihood for women may be due to the obesity paradox, where obese people have been reported to have more favorable cardiovascular outcomes than normal weight people.^24^

Results from the present study show that more women were obese than men (61.9% vrs 31.8%). Furthermore, given that all the women in our study did not have any history of cigarette smoking, it is thus plausible that this translated into a lower risk for them. The ability of the Framingham model to accurately stratify risk as seen in this present study has also been proven in other studies. The AUC obtained in this present study is higher than that reported by other studies where low AUC have been reported in population based for both men and women in Malaysia^23^, Australia^25^, Spain^26^ and Tehran^27^. Unfortunately, there is no report from Africa on the discrimination or calibration of the Framingham risk score. The high AUC observed in our study may therefore be due to that fact that this was a hospital- based study that focused on patients attending a cardiac clinic. This implied a higher likelihood for them to have a higher CVD risk hence the ability of the risk score to better discriminate between low and moderate/high risk.

Our study is limited by being hospital based and so the findings may not necessarily be generalized to the entire population. Additionally, since we solely depended on review of hospital records, many other important potential determinants of CVD risk were not captured.

This study is, however, strengthened by the fact that the use of hospital records enabled us to objectively exclude persons with already established CVD. Another advantage over other studies is minimal misclassification of persons as to their disease status since our population involved patients seen in specialist routine practice.

## Conclusion

Our study has established that majority of the patients attending the cardiac clinics at KATH and Precise Specialist Clinic, Kumasi, Ghana are at moderate to high CVD risk, with men being significantly at higher risk. The Framingham risk score also showed good sensitivity, specificity and discrimination.
